# Dietary Sodium and Potassium Intakes and Kidney Stone Prevalence: The National Health and Nutrition Examination Survey 2011–2018

**DOI:** 10.3390/nu16142198

**Published:** 2024-07-10

**Authors:** Jie Tang, Cara Sammartino, Michel Chonchol

**Affiliations:** 1 Division of Kidney Diseases and Hypertension, Alpert Medical School of Brown University, Providence, RI 02903, USA; 2 Department of Public Health, Johnson & Wales University, Providence, RI 02903, USA; cara.sammartino@jwu.edu; 3 Division of Renal Disease and Hypertension, University of Colorado School of Medicine, Aurora, CO 80045, USA; michel.chonchol@cuanschutz.edu

**Keywords:** kidney stone disease, dietary sodium, dietary potassium

## Abstract

The associations between dietary sodium intake (DSI), dietary potassium intake (DPI), and kidney stone disease (KSD) are not clear. We examined The National Health and Nutrition Examination Survey 2011–2018 to determine the independent associations between daily DSI, DPI, DSI/DPI, and KSD prevalence. In total, 19,405 participants were included for analysis, of which 1,895 had KSD. Higher DSI was not associated with increased odds of KSD in regression analysis when DSI was modeled as a continuous variable (OR = 0.99, 95% CI: 0.99–1.00, *p* = 0.2), or when comparing highest quartile of DSI to lowest quartile (OR = 0.84, 95% CI: 0.68–1.04, *p* = 0.1). Unlike DSI, higher DPI was strongly associated with reduced odds of KSD in regression analysis when DPI was modeled as a continuous variable (OR = 0.99, 95% CI: 0.99–0.99, *p* = 0.02), or when comparing highest quartile of DPI to lowest quartile (OR = 0.75, 95% CI: 0.60–0.94, *p* = 0.01). Lastly, higher DSI/DPI was also strongly associated with increased odds of KSD in regression analysis when DSI/DPI was modeled as a continuous variable (OR = 1.1, 95% CI: 1.01–1.20, *p* = 0.03), or when comparing highest quartile of DPI to lowest quartile (OR = 1.30, 95% CI: 1.10–1.70, *p* = 0.008). All the observed relationships were independent of total calorie intake. In conclusion, both lower DPI and higher DSI/DPI are associated with an increased risk of KSD. Future prospective studies are needed to clarify these causal relationships.

## 1. Introduction

Kidney stone disease (KSD) is common in the US population, with an estimated prevalence of around 10–15% in males and 7–10% in females [1]. It causes significant morbidities and has a huge economic impact on our healthcare system [2]. Kidney stone formation is a complex process with diet playing a crucial role.

Since the vast majority (~80%) of kidney stones are calcium-based, hypercalciuria is considered a major risk factor for kidney stone formation [3]. In healthy volunteers, dietary sodium intake (DSI) has a direct effect on urinary calcium excretion [4,5]. However, its role in promoting kidney stone disease is far from being established. A large population study showed a strong association between DSI and the risk of kidney stone disease [6]. However, this association was not corroborated by other similar population studies [7,8,9]. This inconsistency may reflect the differences in population surveyed and hidden confounders. Unlike dietary sodium, high dietary potassium intake (DPI) was thought to reduce kidney stone risk due to its association with lower urinary calcium excretion [10]. However, results from observational studies have been also conflicting, and there is a lack of interventional trials [7,8,11,12]. Lastly, the balance between dietary sodium and potassium intakes may also be important in modifying kidney stone risk, since both are key determinants of urinary calcium excretion. Unfortunately, to date, the combined effect of DSI and DPI on stone risk remains to be elucidated. Better understanding of these knowledge gaps could guide effective dietary interventions to reduce the burden of KSD. Therefore, in this study of US adults from the National Health and Nutrition Examination Survey (NHANES) 2011–2018, we aim to examine the independent associations of DSI, DPI, and DSI/DPI on the risk of KSD.

## 2. Materials and Methods

### 2.1. Study Population

**Figure 1:**
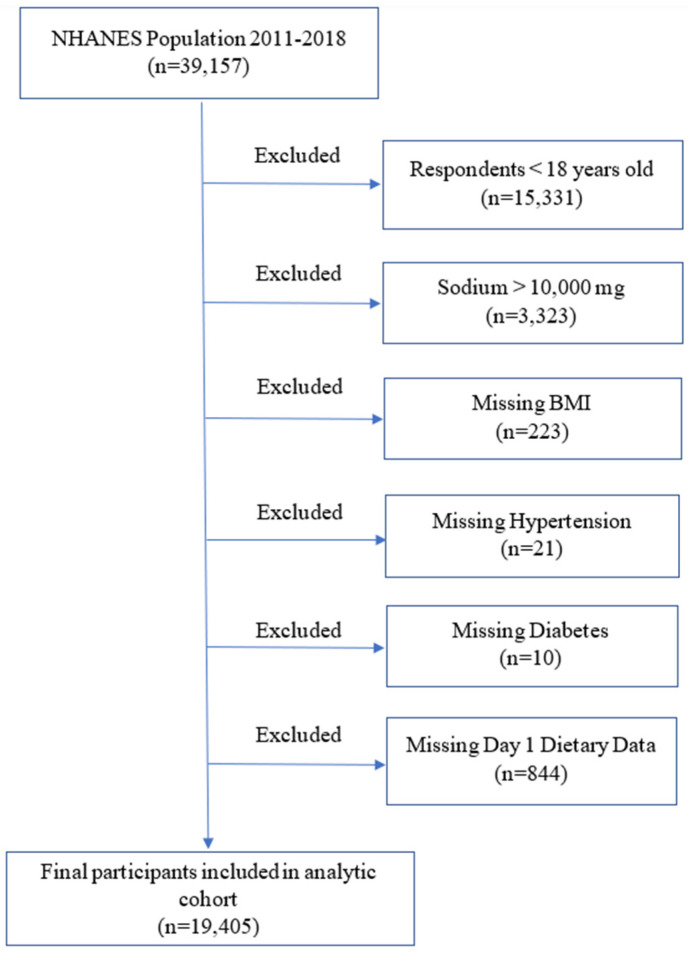
Schematic overview of participant selection and disposition.

The National Health and Nutrition Examination Survey is a national probability sample of the total non-institutionalized civilian population 2 months of age or over in the USA. The survey collected demographic, socioeconomic, dietary, and health-related information, in addition to the examination and laboratory data obtained by highly trained medical personnel. There were a total of 39,157 participants in NHANES 2011–2018, and our analyses were limited to 23,826 adult participants of 18 years or older. The information regarding kidney stone disease prevalence was extracted from the interview data file. ‘Have you ever had a kidney stone?’ was the question asked during the standardized home interview. The adult participants who responded ‘yes’ to the question were considered to have a history of kidney stones. Responders who did not complete at least one dietary recall, had erroneous dietary intake data (DSI > 10,000 mg), had missing body mass index (BMI), had incomplete data on the history of hypertension, diabetes, and diuretics use were excluded (*n* = 4421). Thus, the final sample used in this study included 19,405 adult participants (Figure 1). Among them, 1895 had self-reported history of kidney stones.

### 2.2. Primary Predictors and Outcomes

The primary predictor or independent variables were DSI, DPI, and the ratio of DSI/DPI. In the NHANES, 24 h recall queried all foods/beverages consumed from midnight-to-midnight on the day before the interview. Related nutrient data were obtained from the USDA’s Food and Nutrient Database for Dietary Studies. Mean intake of sodium (mg/day) and potassium (mg/day) were estimated based on dietary recalls in each survey period. The recommended sodium intake was defined as DSI < 2300 mg/day based on the published Dietary Guidelines for Americans 2015–2020 [13]. Optimal DPI recommended by organizations like the World Health Organization was defined at ≥3500 mg per day [14].

The outcome or dependent variable of interest was KSD. It was extracted from the interview data file. ‘Have you ever had a kidney stone?’ was the question asked during the standardized home interview. Adult participants who responded ‘yes’ to the question were considered to have a history of kidney stone disease.

### 2.3. Covariates

Age was defined as age at the time of the interview. Race/ethnicity was self-reported. The history of hypertension was defined as self-reported physician diagnosis of hypertension, or use of any antihypertensives, or if they had mean blood pressure (BP) measurements showing a systolic BP ≥ 140 mm Hg and/or a diastolic BP ≥ 90 mm Hg. The history of diabetes was defined as self-reported physician diagnosis of diabetes or taking hypoglycemics. The history of dyslipidemia was defined as self-reported physician diagnosis of hypercholesterolemia or taking medications to lower blood cholesterol. A history of cardiovascular disease (CVD) was defined as self-reported physician diagnosis of heart failure, coronary heart disease, angina, heart attack or stroke, or having had coronary revascularization. BMI was calculated from the weight and height measured during the physical examination. Hydrochlorothiazide (HCTZ) use was documented during the standardized home interview. Information on cigarette smoking was also collected during the interview. Frequency of alcohol consumption was measured by a food frequency questionnaire. Alcohol consumption (in grams/day) was also evaluated with a 24 h dietary recall and categorized into tertiles of consumption for the purposes of our analysis.

### 2.4. Data Analysis and Statistical Methods

To be included in the study, participants had to have data on kidney stones; dietary sodium; potassium; total calorie intake; age; sex; race; BMI; information regarding thiazide diuretic use; and whether or not they had a history of hypertension, diabetes, and dyslipidemia. The results of continuous variables were expressed as the mean ± standard deviation for normal distributions and as the median for skewed distributions. Categorical variables are expressed as frequencies and percentages. Due to the complex sample strategy of NHANES, appropriate weights and strata were applied. STATA (11.2) PROC SVY:TAB and SVY:MEANS were used to obtain descriptive statistics for the population. Characteristics of the population were compared between kidney stone formers and non-stone formers using the Rao-Scott 2 for the categorical variables and ANOVA for the continuous variables. STATA (11.2) SVY LOGISTIC was used to perform logistic regression to determine if DSI, DPI, and DSI/DPI were associated with a history of kidney stones. Two logistic regression models controlling potential confounders were used to assess the associations in the overall sample. Results are presented as odds ratio (OR) and 95% confidence interval (CI).

### 2.5. Ethical Considerations

Ethical issues were addressed in accordance with the guidelines of the Declaration of Helsinki.

## 3. Results

### Baseline Characteristics

There were a total of 19,405 participants eligible for the final analysis, including 1895 who reported a history of kidney stone disease. Among stone formers, mean DSI was 3438 mg, and only 26% had DSI less than 2300 mg. Mean DPI was 2572 mg and 19% had DPI ≥ 3500 mg. Mean DSI/DPI was 1.5. In comparison, among non-stone formers, mean DSI was 3532 mg (*p* = 0.1) and 24% had DSI less than 2300 mg (*p* = 0.2); mean DPI was 2665 mg (*p* = 0.03) with 21% having DPI ≥ 3500 mg (*p* = 0.2); DSI/DPI was 1.4 (*p* = 0.2). As shown in Table 1, stone formers tended to be older, males, and non-Hispanic whites, and to have a higher BMI compared to non-stone formers. They were also more likely to have hypertension, diabetes, dyslipidemia, cardiovascular disease, and take thiazide diuretics, and were more likely to be an active smoker and heavy drinker.

When we break down DSI, DPI, and DSI/DPI into quartiles, participants in the highest quartile of DSI were more likely to be younger, to be male and non-Hispanic whites, to have a higher BMI, to have lower prevalence of hypertension, dyslipidemia, and cardiovascular disease, and to be less likely to be an active smoker and to drink alcohol heavily than participants in the lower quartiles of DSI (Table 2). Participants in the highest quartile of DPI were more likely to be older, to be male and non-Hispanic whites, to have a lower BMI, and to have dyslipidemia, but were less likely to have a history of diabetes or cardiovascular disease, to be an active smoker, and to drink heavily than participants in the lower quartiles of DPI (Table 3). With regard to DSI/DPI, participants in the highest quartiles were more likely to be younger, to be male, to have a higher BMI, to be an active smoker, and to drink alcohol heavily, but were less likely to be non-Hispanic whites or to have histories of hypertension, dyslipidemia, or cardiovascular disease (Table 4).

Dietary Sodium and Potassium Intakes and Kidney Stone Prevalence.

The logistic regression analyses examining the relationships between DSI, DPI, DSI/DPI, and kidney stone disease are shown in Table 5 and Table 6. Higher sodium intake was not associated with increased odds of kidney stone disease in univariate analysis (OR = 0.99, 95% CI: 0.99–1.00, *p* = 0.1), or in regression analysis when DSI was modeled as a continuous variable after adjustment for age, sex, race, BMI, histories of hypertension, diabetes, dyslipidemia, cardiovascular disease, usage of HCTZ, cigarette smoking, and alcohol drinking (OR = 0.99, 95% CI: 0.99–1.00, *p* = 0.2). When kidney stone risk was examined with extreme categories of DSI or by binary comparison of DSI, the multivariate-adjusted OR for stone formation was 0.84 (95% CI: 0.68–1.04, *p* = 0.1) in those who consumed >4321 mg/day DSI compared to those with <2227 mg/day of DSI, and the OR was 1.10 (95% CI: 0.93–1.20, *p* = 0.3) in those who consumed ≤2300 mg DSI compared to those with >2300 mg DSI (Table 5). When we adjusted for dietary calorie intake using the ratio of DSI over dietary calorie intake (DCI) as the predictor variable, the findings were similar (Supplemental Table S1). Unlike DSI, higher DPI was associated with reduced odds of kidney stone disease in both univariate analysis (OR = 0.99, 95% CI: 0.99–0.99, *p* = 0.04) and in regression analysis after adjustment for age, sex, race, BMI, histories of hypertension, diabetes, dyslipidemia, cardiovascular disease, usage of HCTZ, cigarette smoking, and alcohol drinking when DPI was modeled as a continuous variable (OR = 0.99, 95% CI: 0.99–0.99, *p* = 0.02). When we examined the extreme categories of DPI, there was a 25% reduction in stone risk (multivariate-adjusted OR = 0.75, 95% CI: 0.60–0.94, *p* = 0.01) in those who consumed >3209 mg/day DPI compared to those with <1699 mg/day of DPI (Table 5). When we adjusted DCI using DPI/DCI as the predictor variable, the findings were also similar (Supplemental Table S1). Lastly, when we examined the combined effect of DSI and DPI reflected by DSI/DPI, higher DSI/DPI was associated with increased odds of kidney stone disease in both univariate analysis (OR = 1.2, 95% CI: 1.10–1.20, *p* = 0.04) and in regression analysis after adjustment for age, sex, race, BMI, histories of hypertension, diabetes, dyslipidemia, cardiovascular disease, usage of HCTZ, cigarette smoking, and alcohol drinking when DSI/DPI was modeled as a continuous variable (OR = 1.1, 95% CI: 1.01–1.20, *p* = 0.03). When we examined the extreme categories of DSI/DPI, there was a 30% increase in stone risk (multivariate-adjusted OR = 1.30, 95% CI: 1.10–1.70, *p* = 0.008) when quartile 4 was compared to quartile 1, with a gradient effect observed in quartile comparisons (Table 5).

There were no interaction effects of dietary intake X on other covariates in the regression model on kidney stone formation. In our multivariate logistic regression analyses, the following variables were found to have significant associations with increased odds of kidney stone disease: older age, male sex, non-Hispanic white, increasing BMI, history of hypertension, diabetes and dyslipidemia, active cigarette smoking, and heavy alcohol use (Table 6).

## 4. Discussion

In this large US population based cross-sectional study, we showed that DSI was not associated with an increased risk of kidney stone disease. However, lower DPI and higher DSI/DPI significantly increased the risk of kidney stones. The results of this large population study highlights the significance of DPI in kidney stone management. To our knowledge, this is the first study examining the combinational effect of DSI and DPI on the risk of kidney stone disease.

Both sodium and potassium are key nutrients essential for maintenance of body fluid volume, acid and electrolyte balance, and normal cellular function. In modern society, dietary sodium and potassium content have been markedly altered. Food processing increases the sodium and reduces the potassium content of food [15]. As a result, the majority of adult populations worldwide have mean sodium intakes above 2300 mg per day [16,17], and potassium intakes below 3500 mg per day [17,18]. Higher DSI is directly associated with hypercalciuria, a major risk factor for KSD. The association between DSI and urinary calcium excretion was first observed in an animal model by Walser et al. [19], and was later confirmed in human studies [20,21,22]. This correlation between urine sodium and calcium remained significant even during fasting, and dietary salt restriction lowered urine calcium excretion, further proving a causal relationship [23]. Given that hypercalciuria is a strong risk factor for kidney stone disease, it is only natural to assume that dietary salt restriction would be beneficial for stone prevention. However, large population studies showed conflicting data on the association between DSI and incident kidney stone risk [6,7,8,9]. These discrepancies may reflect the difficulties in obtaining reliable estimates of dietary salt intake and the presence of many hidden confounders. In this study of a large nationally representative sample of the US population, we also failed to show a significant association between DSI and kidney stone disease, regardless of DSI being analyzed as a continuous or as a categorical variable. To account for variations in calorie consumption, we also used DSI/DCI as a predictor variable, a measure that has been well validated in clinical trials [24]. Again, we did not show any association of sodium-dense diet on kidney stone formation, regardless of the analytical approaches. We believe our results are consistent with higher DSI not increasing the risk of kidney stone formation. Although not proven, it is conceivable that higher DSI promotes oral hydration, and therefore offsets its harmful effect of raising urine calcium excretion. Indeed, a retrospective analysis of 880 kidney stone formers revealed that higher urine sodium led to an increase in urine calcium, but more importantly also a large increase in urine volume, resulting in a significant reduction in calcium oxalate supersaturation in the urine [25].

Contrary to sodium, adequate body potassium store is essential for the renal tubular reabsorption of calcium, therefore can reduce urinary calcium excretion. DPI has been shown to modulate sodium-chloride cotransporter channel activity via basolateral Kir4.1 at the distal convoluted tubule [26]. As a result, higher DPI can decrease urinary calcium excretion by inducing transient sodium diuresis, which results in a temporary contraction of the extracellular fluid volume and an increase in renal tubular calcium reabsorption. Furthermore, potassium increases renal phosphate reabsorption and reduces vitamin D due to phosphate retention, resulting in decreased intestinal calcium absorption and subsequent urinary calcium excretion [27]. Indeed, a human study did confirm the inverse relationship between urinary potassium and calcium excretions [28]. An interventional study further demonstrated that potassium administration reduces and potassium deprivation increases urinary calcium excretion in healthy adults [10]. Lastly, potassium reduces the expression of Na^+^-dicarboxylate cotransporter 1, a key protein involved in renal citrate reabsorption [29]. In a cohort of recurrent calcium kidney stone formers, urine potassium had a strong positive correlation with urine citrate level, and dietary potassium chloride supplementation resulted in a significant increase in urinary citrate excretion [30]. Therefore, inadequate DPI can stimulate renal tubular citrate reabsorption resulting in hypocitraturia, another major risk factor for calcium stone formation [31]. To demonstrate a direct effect of DPI on the overall stone risk, we showed a strong independent effect of DPI on the risk of kidney stone in this large US population cohort. This effect remained strongly significant after we adjusted DCI using DPI/DCI as the predictor variable. Our finding is consistent with another large population study which revealed a significantly lowered urinary potassium excretion among incidence kidney stone formers, indicating an important role of DPI in kidney stone formation [32].

In addition to their individual effects, the combined activity of dietary sodium and potassium is worth investigating, as recent clinical studies suggested that DSI/DPI ratio was more predictive of clinical outcomes (i.e., hypertension, cardiovascular disease event) than either DSI or SPI alone [33,34]. Urine sodium/potassium ratio also appears to be associated more strongly with urinary calcium excretion than either urine sodium or potassium alone [28], indicating an additive or even synergistic effect of high DSI plus low DPI in promoting hypercalciuria. Indeed, we found that higher DSI/DPI ratio was strongly associated with kidney disease, an effect much stronger than either the DSI or DPI alone. This finding is novel, because for the first time it provides direct evidence that dietary salt restriction combined with an increased DPI could be more effective for kidney stone prevention.

Our study has limitations worth noting. First, as a cross-sectional study, the present analysis is limited in its ability to establish causal or temporal relationships between dietary sodium and potassium intake and kidney stone formation. Second, our dietary estimates excluded sodium from table salt, supplements, and medications, which account for an estimated 6% of total dietary sodium intake [35], thus underestimating the proportion of participants with excessive intakes. We also excluded potassium from salt substitutes, supplements, and medications. Although it is possible that stone formers in this cohort were following a low sodium diet after the publication of an AUA guideline in 2014 recommending dietary salt restriction for patients with KSD [36], when we examined dietary intakes before and after 2014, no change in DSI was found among stone formers in this large US population cohort (Supplemental Table S2). It is also unlikely that stone formers would increase DPI or take a potassium supplement for secondary stone prevention, since there is a lack of clinical evidence so far to support such practice. However, the potential confound of the use of potassium citrate, a medication commonly prescribed for KSD, needs to be considered. Unfortunately, information regarding such medication use is lacking in NHANES. Third, due to the high prevalence of hypertension and CVD, stone formers may restrict DSI as a result, therefore introducing a potential bias. To address this concern, we performed sensitivity analysis excluding participants with hypertension and CVD, and found almost identical results. Fourth, the daily nutrient intakes were calculated from dietary recall, which may not reflect habitual intake. Additionally, the differences of DSI and DPI between stone formers and non-stone formers were small, although significant. Fifth, the kidney stone cases were self-reported, and some participants may have kidney stone disease without self-awareness or clinical diagnosis. This may have led to potential misclassification, but should be non-selective with regard to different dietary intake. Furthermore, if this misclassification exists, the results should be biased toward null. Finally, we do not have information on stone composition, although 80% of kidney stones in the general population like NHANES are calcium based.

## 5. Conclusions

In summary, although DSI by itself was not a strong predictor of kidney stone disease, higher DSI combined with lower DPI did have a strong association with kidney stone risk. Regardless of DSI, lower DPI was strongly associated with increased odds of kidney stone disease.

Rising kidney stone prevalence world-wide showcases the failure to implement effective interventions in the management of KSD, calling for more effective public health policy to reduce the disease burden on a population level. Public health interventions aimed at reducing DSI and increasing DPI may therefore be potential cost-effective measures for reducing the burden of KSD.

## Figures and Tables

**Table 1:** Basic characteristics of the study population.

		Stone Former	Non-Stone Former	*p* Value
Total number		1895	17510	
Age (years)		54 (±0.4)	47 (±0.3)	<0.001
Sex (male %)		985 (52)	8230 (47)	0.01
Race (Non-Hispanic White %)		1421 (75)	11,382 (65)	<0.001
BMI (mg/m^2^)		30.9 (±0.2)	29.2 (±0.1)	<0.001
History of hypertension (%)		910 (48)	5603 (32)	<0.001
History of diabetes (%)		360 (19)	1576 (9)	<0.001
History of dyslipidemia (%)		872 (46)	5778 (33)	<0.001
Cardiovascular disease (%)		284 (15)	1401 (8)	<0.001
Thiazide use (%)		208 (11)	1226 (7)	<0.001
Smoking (%)	Everyday/some day	379 (20)	3152 (18)	<0.001
	Past smoker	569 (30)	4202 (24)	
	Not at all	948 (50)	10,156 (58)	
Alcohol (%)	Heavy	38 (2)	1051 (6)	0.001
	Light	360 (19)	3852 (22)	
	None	1477 (78)	12,607 (72)	
DSI	Mean (mg)	3438 (±56)	3532 (±17)	0.1
	<2300 mg/d (%)	493 (26)	4202 (24)	0.2
DPI	Mean (mg)	2572 (±43)	2665 (±17)	0.03
	≥3500 mg/d (%)	360 (19)	3677 (21)	0.2
DSI/DPI		1.5 (±0.03)	1.4 (±0.01)	0.2

Values are expressed as means (±SE) or numbers (%). Abbreviations: BMI, body mass index. DSI, dietary sodium intake. DPI, dietary potassium intake.

**Table 2:** Quartiles of DSI in the whole NHANES 2011–2018 cohort.

		DSI (mg/Day)				*p* Value
		0–2226	2227–3149	3150–4321	>4321	
Age (years)		50 (±0.4)	49 (±0.4)	47 (±0.4)	43 (±0.4)	<0.0001
Sex (male %)		1538 (30)	1999 (39)	2566 (50)	3634 (71)	<0.0001
Race (Non-Hispanic White %)		3230 (63)	3434 (67)	3438 (67)	3327 (65)	0.0003
BMI (kg/m^2^)		28.8 (±0.2)	29.1 (±0.2)	29.2 (±0.2)	29.8 (±0.2)	<0.0001
History of hypertension (%)		1846 (36)	1691 (33)	1642 (32)	1638 (32)	0.0003
History of diabetes (%)		564 (11)	564 (11)	462 (9)	461 (9)	0.1
History of dyslipidemia (%)		1794 (35)	1794 (35)	1642 (32)	1587 (31)	0.01
Cardiovascular disease (%)		564 (11)	513 (10)	411 (8)	307 (6)	<0.0001
Thiazide use (%)		410 (8)	410 (8)	359 (7)	358 (7)	0.3
Smoking (%)	Active	1025 (20)	820 (16)	872 (17)	972 (19)	<0.0001
	Past smoker	1128 (22)	1230 (24)	1334 (26)	1280 (25)	
	Not at all	2974 (58)	3024 (59)	2925 (57)	2866 (56)	
Alcohol (%)	Heavy	3999 (78)	3844 (75)	3695 (72)	3634 (71)	<0.0001
	Light	974 (19)	1128 (22)	1180 (23)	1126 (22)	
	None	154 (3)	154 (3)	257 (5)	205 (4)	

Values are expressed as means (±SE) or numbers (%). Abbreviations: BMI, body mass index. DSI, dietary sodium intake.

**Table 3:** Quartiles of DPI in the whole NHANES 2011–2018 cohort.

		DPI (mg/Day)				*p* Value
		0–1698	1699–2374	2375–3209	>3209	
Age (years)		45 (±0.5)	47 (±0.5)	48 (±0.4)	48 (±0.4)	0.0003
Sex (male %)		1693 (33)	2049 (40)	2462 (48)	3481 (68)	<0.0001
Race (Non-Hispanic White %)		3027 (59)	3278 (64)	3540 (69)	3532 (69)	<0.0001
BMI (kg/m^2^)		29.8 (±0.2)	29.2 (±0.2)	29.3 (±0.2)	28.7 (±0.2)	0.0001
History of hypertension (%)		1744 (34)	1741 (34)	1693 (33)	1638 (32)	0.6
History of diabetes (%)		616 (12)	512 (10)	513 (10)	461 (9)	0.0006
History of dyslipidemia (%)		1539 (30)	1741 (34)	1744 (34)	1792 (35)	0.0002
Cardiovascular disease (%)		564 (11)	461 (9)	410 (8)	410 (8)	0.0002
Thiazide use (%)		359 (7)	410 (8)	410 (8)	358 (7)	0.4
Smoking (%)	Active	1180 (23)	973 (19)	821 (16)	870 (17)	<0.0001
	Past smoker	975 (19)	1127 (22)	1334 (26)	1485 (29)	
	Not at all	2975 (58)	3022 (59)	2975 (58)	3020 (59)	
Alcohol (%)	Heavy	4258 (83)	3893 (76)	3642 (71)	3532 (69)	<0.0001
	Light	770 (15)	1076 (21)	1283 (25)	1229 (24)	
	None	103 (2)	154 (3)	205 (4)	870 (17)	

Values are expressed as means (±SE) or numbers (%). Abbreviations: BMI, body mass index. DPI, dietary potassium intake.

**Table 4:** Quartiles of DSI/DPI in the whole NHANES 2011–2018 cohort.

		DSI/DPI				*p* Value
		<1.01	1.01–1.35	1.35–1.77	>1.77	
Age (years)		52 (±0.4)	49 (±0.4)	46 (±0.4)	41 (±0.4)	<0.0001
Sex (male %)		2181 (43)	2477 (48)	2553 (50)	2686 (52)	<0.0001
Race (Non-Hispanic White %)		3500 (69)	3457 (67)	3369 (66)	3100 (60)	<0.0001
BMI (kg/m^2^)		28.1 (±0.2)	28.9 (±0.2)	29.8 (±0.2)	30.2 (±0.2)	<0.0001
History of hypertension (%)		1724 (34)	1754 (34)	1736 (34)	1601 (31)	0.05
History of diabetes (%)		507 (10)	568 (11)	511 (10)	517 (10)	0.6
History of dyslipidemia (%)		1877 (37)	1858 (36)	1685 (33)	1395 (27)	<0.0001
Cardiovascular disease (%)		558 (11)	464 (9)	408 (8)	362 (7)	0.001
Thiazide use (%)		406 (8)	413 (8)	408 (8)	362 (7)	0.6
Smoking (%)	Active	862 (17)	826 (16)	919 (18)	1137 (22)	<0.0001
	Past smoker	1319 (26)	1393 (27)	1225 (24)	1033 (20)	
	Not at all	2891 (57)	2941 (57)	2961 (58)	2996 (58)	
Alcohol (%)	Heavy	3601 (71)	3767 (73)	3727 (73)	4081 (79)	<0.0001
	Light	1217 (24)	1135 (22)	1123 (22)	930 (18)	
	None	254 (5)	258 (5)	204 (4)	207 (4)	

Values are expressed as means (±SE) or numbers (%). Abbreviations: BMI, body mass index. DSI, dietary sodium intake. DPI, dietary potassium intake.

**Table 5:** OR of kidney stone according to DSI and DPI as continuous, categorical, or binary variables in the multivariate regression model.

		OR (95% CI)	*p* Value
DSI			
Continuous variable		0.99 (0.99–1.00)	0.2
Categorial variable	Quartile 4 vs. 1	0.84 (0.68–1.04)	0.1
	Quartile 3 vs. 1	1.05 (0.85–1.30)	0.6
	Quartile 2 vs. 1	0.95 (0.79–1.10)	0.6
≤2300 mg vs. >2300 mg		1.10 (0.93–1.20)	0.3
DPI			
Continuous variable		0.99 (0.99–0.99)	0.02
Categorial variable	Quartile 4 vs. 1	0.75 (0.60–0.94)	0.01
	Quartile 3 vs. 1	0.82 (0.67–1.01)	0.06
	Quartile 2 vs. 1	0.82 (0.68–0.97)	0.02
>3500 mg vs. ≤3500 mg		0.87 (0.72–1.04)	0.1
DSI/DPI			
Continuous variable		1.10 (1.01–1.20)	0.03
Categorial variable	Quartile 4 vs. 1	1.30 (1.10–1.70)	0.008
	Quartile 3 vs. 1	1.20 (0.99–1.40)	0.06
	Quartile 2 vs. 1	1.20 (0.91–1.50)	0.2
>0.6 vs. ≤0.6		1.20 (0.86–1.67)	0.3

Abbreviations: DSI, dietary sodium intake. DPI, dietary potassium intake.

**Table 6:** Multivariate-adjusted OR of covariates from the model with dietary intakes as continuous variables.

	DSI		DPI		DSI/DPI	
	OR (95% CI)	*p* Value	OR (95% CI)	*p* Value	OR (95% CI)	*p* Value
Age (years)	1.02 (1.01–1.02)	<0.001	1.02 (1.01–1.02)	<0.001	1.02 (1.01–1.02)	<0.001
Sex (Male)	1.20 (1.04–1.50)	0.02	1.30 (1.10–1.50)	0.005	1.2 (1.01–1.40)	0.03
Race (White)	2.30 (1.90–2.70)	<0.001	2.30 (2.00–2.70)	<0.001	2.3 (1.97–2.70)	<0.001
BMI (>30 kg/m^2^)	1.70 (1.40–1.96)	<0.001	1.60 (1.40–1.90	<0.001	1.6 (1.40–1.90)	<0.001
History of hypertension	1.30 (1.04–1.50)	0.02	1.20 (1.03–1.50)	0.02	1.2 (1.03–1.50)	0.02
History of diabetes	1.50 (1.20–1.80)	<0.001	1.50 (1.20–1.80)	<0.001	1.5 (1.20–1.80)	<0.001
History of dyslipidemia	1.20 (1.01–1.30	0.03	1.20 (1.01–1.30	0.03	1.2 (1.03–1.50)	0.03
Cardiovascular disease	1.20 (0.92–1.50)	0.2	1.1 (0.91–1.50)	0.3	1.2 (0.92–1.50)	0.2
Thiazide use	1.10 (0.86–1.30)	0.6	1.1 (0.85–1.30)	0.6	1.05 (0.85–1.30)	0.6
Smoking (active)	1.30 (1.10–1.50)	0.01	1.3 (1.04–1.50)	0.02	1.3 (1.10–1.50)	0.01
Alcohol (heavy)	0.49 (0.32–0.76)	0.002	0.51 (0.33–0.78)	0.002	0.5 (0.33–0.76)	0.002
DSI	0.99 (0.99–1.00)	0.2	N/A	N/A	N/A	N/A
DPI	N/A	N/A	0.99 (0.99–0.99)	0.02	N/A	N/A
DSI/DPI	N/A	N/A	N/A	N/A	1.1 (1.01–1.20)	0.03

N/A: not applicable. Abbreviations: BMI, body mass index. DSI, dietary sodium intake. DPI, dietary potassium intake.

## Data Availability

Records and data pertaining to this study are stored electronically at the Division of Kidney Diseases and Hypertension, Alpert Medical School of Brown University in Providence, USA, and can be provided by the corresponding author upon reasonable request.

## Supplementary Materials

The following supporting information can be downloaded at: https://www.mdpi.com/article/10.3390/nu16142198/s1, Table S1. Odds ratio of prevalent kidney stone according to DSI/DCI & DPI/DCI as continuous, categorical or binary variables in the multivariate regression model. Table S2. Linear regression models evaluating the differences in DSI among kidney stone formers comparing 2015-2018 vs. 2011–2014.
