# Supplementary material for: Dietary Sodium and Potassium Intakes and Kidney Stone Prevalence: The National Health and Nutrition Examination Survey 2011–2018
Source: Nutrients. 2024 Jul 10;16(14):2198. doi: 10.3390/nu16142198 (PMC11279477; doi:10.3390/nu16142198)
Supplement: Supplementary file 1 [file nutrients-16-02198-s001.zip › nutrients-3095096-supplementary.pdf]

Supplementary Materials: Supplemental Table S1. Odds ratio of prevalent kidney stone according to DSI/DCI & DPI/DCI as continuous, categorical or binary variables in the multivariate regression model

		OR (95% confidence interval)	P value
DSI/DCI			
Continuous variable		0.96 (0.84-1.10)	0.5
Categorial variable	Quartile 4 vs. 1	0.90 (0.72-1.10)	0.3
	Quartile 3 vs. 1	0.80 (0.67-0.94)	0.01
	Quartile 2 vs. 1	1.00 (0.80-1.20)	1.0
>1.1 vs. ≤ 1.1 mg/kcal		0.90 (0.71-1.10)	0.4
DPI/DCI			
Continuous variable		0.75 (0.64-0.87)	<0.001
Categorial variable	Quartile 4 vs. 1	0.71 (0.58-0.86)	0.001
	Quartile 3 vs. 1	0.78 (0.64-0.96)	0.02
	Quartile 2 vs. 1	0.87 (0.73-1.00)	0.1
>1.7 vs. ≤ 1.7 mg/kcal		0.70 (0.56-0.87)	0.002

Abbreviations: DSI, dietary sodium intake. DPI, dietary potassium intake. DCI, dietary calorie intake.

Supplemental Table S2. Linear regression models evaluating the differences in DSI among kidney stone formers comparing 2015-2018 vs. 2011-2014

DSI	Univariate		Multivariate	
	β (SE)	P value	β (SE)	P value
2011-2014	REF		REF	
2015-2018	120.10 (111.70)	0.3	127.30 (109.05)	0.3

SE= standard error. Multivariate regression model adjusted for age, sex, race, BMI, histories of hypertension, diabetes, dyslipidemia, cardiovascular disease, usage of thiazide, cigarette smoking and alcohol drinking. Abbreviation: DSI, dietary sodium intake.
